# Urine 11-Dehydro-Thromboxane B2 in Aspirin-Naive Males with Metabolic Syndrome

**DOI:** 10.3390/jcm11123471

**Published:** 2022-06-16

**Authors:** Wiesław Piechota, Paweł Krzesiński, Katarzyna Piotrowicz, Grzegorz Gielerak, Małgorzata Kurpaska, Alicja Rączka, Agnieszka Woźniak-Kosek

**Affiliations:** 1 Department of Laboratory Diagnostics, Military Institute of Medicine, Szaserów Street 128, 04-141 Warsaw, Poland; wpiechota@wim.mil.pl (W.P.); araczka@wim.mil.pl (A.R.); awozniak-kosek@wim.mil.pl (A.W.-K.); 2 Department of Cardiology and Internal Diseases, Military Institute of Medicine, Szaserów Street 128, 04-141 Warsaw, Poland; kpiotrowicz@wim.mil.pl (K.P.); ggielerak@wim.mil.pl (G.G.); mkurpaska@wim.mil.pl (M.K.)

**Keywords:** urine 11-dehydro-thromboxane B2, metabolic syndrome, platelet activity, risk factor, aspirin

## Abstract

Urine 11-dehydro-thromboxane B2 (11-dehydro-TXB2), an indirect measure of platelet activity, is elevated in cardiovascular diseases and diabetes. The purpose of our study was to determine whether urine 11-dehydro-TXB2 is elevated in aspirin-naive males with metabolic syndrome (MS) and to determine predictors of 11-dehydro-TXB2 levels. The secondary aim was to evaluate whether these MS patients could be potential candidates for the aspirin-mediated prevention of atherosclerotic cardiovascular diseases (ASCVDs). In 82 males with MS (76 hypertensive), anthropometric measures, urine 11-dehydro-TXB2, platelet count, creatinine, glucose, insulin, estimated insulin resistance, lipid parameters, high-sensitivity C-reactive protein (hs-CRP), adiponectin, homocysteine, and ten-year risk of fatal cardiovascular disease (SCORE) were assessed. Urine 11-dehydro-TXB2 levels were elevated (≥2500 pg/mg creatinine) in two-thirds of patients, including 11 high-risk patients (SCORE ≥ 5%). Homocysteine, adiponectin, hs-CRP, waist-to-hip ratio, and total cholesterol were found to be predictors of urine 11-dehydro-TXB2. In conclusion, there is a high incidence of elevated urine 11-dehydro-TXB2 in males with MS, including in some patients who are at a high or very high risk of ASCVDs. 11-dehydro-TXB2 levels are associated with hyperhomocysteinemia, inflammation, fat distribution, hypercholesterolemia, and adiponectin concentrations. Elevated 11-dehydro-TXB2 levels may support the use of personalised aspirin ASCVD prevention in high-risk males with MS. Giuseppe Patti.

## 1. Introduction

Urine 11-dehydro-thromboxane B2 (11-dehydro-TXB2) is a major final metabolite of thromboxane A2 (TXA2) which is responsible for platelet activation and irreversible aggregation. The intermediate product of this pathway is inactive thromboxane B2 (TXB2), which is further converted into 2,3-dinor-TXB2 and 11-dehydro-TXB2 and excreted in urine [1]. As most TXA2 is released from platelets, its stable metabolite, urine 11-dehydro-TXB2, is regarded as a reliable index of in vivo platelet activity. The increased activity and reactivity of platelets to physiological and exogenous agonists are strongly involved in the pathogenesis of atherosclerotic and atherothrombotic diseases [2]. Therefore, measures of curtailing platelet activity were implemented in the primary and secondary prevention of these diseases. However, there are still doubts about the rationale and benefits of aspirin in primary prevention [3,4]. The general recommendation is to avoid using aspirin in the primary prevention of atherosclerotic cardiovascular diseases (ASCVDs), because the risk of major bleeding may outweigh the cardioprotective benefits of aspirin therapy. The guidelines directly referring to aspirin use in metabolic syndrome (MS) preclude such a strategy in standard practice [5]. However, aspirin may be considered for primary prevention in diabetic patients at high or very high risk [6]. Moreover, in some non-diabetic patients with established ASCVD for whom a high or very high cardiovascular risk cannot be ruled out, the benefits of aspirin may outweigh the risks [7]. Platelet activity has been reported to be increased in obesity [8] and MS [9,10], but the data in this field of research are insufficient. The final decision on whether to consider aspirin use in high-risk patients with MS may be supported by the assessment of basal platelet activity in conjunction with ASCVD risk.

The aim of our study was to assess the platelet activity in aspirin-naive patients with MS by measuring urine 11-dehydro-TXB2. An additional aim was to determine whether 11-dehydro-TXB2 levels were correlated with well-known risk factors for cardiovascular disease and diabetes and to determine the potential predictors of 11-dehydro-TXB2 levels. Thirdly, based on our study group, we tried to determine whether there are any MS patients in whom ASCVD aspirin prevention may be conditionally considered.

## 2. Methods

### 2.1. Study Group

This study included 82 consecutive male patients with MS diagnosed in ambulatory settings according to the International Diabetes Federation (IDF) criteria [11]. They were recruited to the clinical research study (stationary grant no. 69/WIM) designed to perform their detailed clinical evaluation, including, i.e., a wide set of metabolic parameters and cardiac haemodynamics. The subjects were admitted to the Department of Cardiology and Internal Medicine (Military Institute of Medicine in Warsaw, Poland) between 2009 and 2013. The patients had no history of diabetes, chronic kidney disease, thyroid dysfunction, or cardiovascular disease (except for arterial hypertension). Any symptoms suggesting cardiovascular disease (including dyspnoea, chest pain, palpitations) were also exclusion criteria. The anthropometric parameters including waist circumference (WC), body mass index (BMI), and waist-to-hip ratio (WHR) were measured according to standard methods [12]. The study was approved by the local ethics committee (no. 44/WIM/2010). Each patient provided written informed consent to participate in the study.

### 2.2. Basic Clinical Assessments

Office blood pressure (systolic blood pressure (SBP) and diastolic blood pressure (DBP)) measurements were performed according to the 2017 American College of Cardiology (ACC)/American Heart Association (AHA) Hypertension Guidelines [13]. Based on medical history, the physical examination, and the results of previous or current laboratory tests, the cardiovascular risk SCORE (systemic coronary risk evaluation) was calculated for each patient (10-year risk of a fatal cardiovascular event) [14].

### 2.3. Laboratory Tests

Urine samples for 11-dehydro-TXB2 determination were collected in the morning and immediately sent to the laboratory, where after centrifugation, supernatants were frozen until the time of analysis (within one month). Before analysis, they were thawed and centrifuged, and the supernatants were used for analyses. The quantification of 11-dehydro-TXB2 was performed using an enzyme-linked immunosorbent assay (ELISA) method using the AspirinWorks^®^ (11Dehydro Thromboxane B_2_) kit manufactured by Corgenix Inc. (Broomfield, CO, USA). The results were standardised by expressing them relative to urine creatinine (i.e., in pg of 11-dehydro-TXB2 per mg of creatinine). Urinary 11-dehydro-TXB2 levels were stratified according to the algorithm by Lopez et al. [2]. Specifically, the 11-dehydro-TXB2 levels below 2500 pg/mg creatinine corresponded to normal baseline (aspirin-free) platelet activity, and the two other strata corresponded to moderate and high activity. When 11-dehydro-TXB2 is over 2500 pg/mg creatinine, cardiovascular disease risk assessment is recommended, and if the risk is indeed elevated, aspirin is prescribed. In patients with the highest levels of 11-dehydro-TXB2 (>10,000 pg/mg creatinine), the evaluation of cardiovascular disease risk factors and the aspirin response test should be performed (with a cut-off of 1500 pg/mg creatinine indicating a positive response).

After overnight fasting, blood samples were collected by venipuncture into glass tubes containing K_3_EDTA (for platelet count and glycated haemoglobin (HbA1c) determination) and tubes without anticoagulants (for the clinical chemistry analytes). Samples in the latter tubes were centrifuged at 2000× *g* for 15 min at 4 °C to obtain serum. Platelet counts were estimated with a Pentra DX 120 Horiba ABX automatic haematology analyser. Creatinine, fasting glucose (FG), total cholesterol (TC), high-density lipoprotein cholesterol (HDL-C), triglycerides (TG), and homocysteine were determined in serum by standard methods using Roche Diagnostics reagents. Low-density lipoprotein cholesterol (LDL-C) was calculated according to the Friedewald equation [15]; in cases with TG above 400 mg/dL, the LDL-C levels were calculated using the method described by Martin et al. [16] and implemented in the Johns Hopkins Medicine LDL Calculator [17]. Insulin, apolipoprotein B (apoB), and HbA1c were determined with the Roche Cobas 6000 analyser by electrochemiluminescence immunoassay and immunoturbidimetric methods, respectively. C-reactive protein (hs-CRP) concentrations were assessed by a highly sensitive immunonephelometric assay using Siemens reagents (CardioPhase^®^ hsCRP) and a BN2 analyser from the same manufacturer. Serum adiponectin concentrations were determined using the Human Adiponectin Platinum ELISA reagent kits produced by eBioscience. Estimated insulin resistance was quantified according to the homeostatic model assessment (HOMA) proposed by Matthews using the following equation: HOMA-IR = [insulin (µU/mL) × glucose (mg/dL)]/405 [18]. The cut-off used for insulin resistance was an upper limit of the third quartile of HOMA-IR, equal to 3.4, established for the Polish population [19]. The estimated glomerular filtration rate (eGFR) was calculated according to the Modification of Diet in Renal Disease (MDRD) formula [20].

### 2.4. Statistical Analysis

Statistical analyses were performed with Statistica for Windows, Version 12 (StatSoft, Inc., Tulsa, OK, USA). The data normality was checked with visual inspection and the Kolmogorov–Smirnov test. Data were expressed as means and standard deviations or medians with ranges. The Mann–Whitney U test was used to assess the differences between subgroups of patients for variables with non-Gaussian distributions. Otherwise, the Student’s t-test was used. In addition, logarithmic transformation was applied to the positively skewed data. Spearman or Pearson correlation coefficients were calculated for selected variables. Multiple linear regression was used to assess the factors independently associated with 11-dehydro-TXB2 levels. A two-sided *p*-value < 0.05 was considered significant.

## 3. Results

### 3.1. Basic Characteristics

The mean age of the study group was 43.3 ± 9.7 years. Nearly all patients (*n* = 76, 93%) had arterial hypertension, and 53 (65%) were treated with antihypertensive drugs. Twenty-eight percent of the patients (*n* = 23) were treated with statins. None of the patients had taken aspirin for 2 weeks preceding the testing. The detailed basic characteristics of the study group are presented in Table 1 and Table 2.

### 3.2. Urinary 11-Dehydro-TXB2 Assessment

The frequency distribution of urinary 11-dehydro-TXB2 levels in MS patients, despite some skewness, was close to normal. Table 3 contains the 11-dehydro-TXB2 results for all patients, as well as the results stratified according to the algorithm proposed by Lopez et al. [2] and the CVD risk calculated by the European Society of Cardiology (ESC) SCORE. Among the patients with moderately to highly elevated levels of 11-dehydro-TXB2, 11 (20%) had a high CVD risk (SCORE ≥ 5%). One-third of the subjects (27) had 11-dehydro-TXB2 levels of less than 2500 pg/mg creatinine, among which 10 had levels below 1500 pg/mg creatinine.

### 3.3. Urinary 11-Dehydro-TXB2 Correlations

Among the basic clinical parameters, only WHR was weakly correlated with 11-dehydro-TXB2, but this association did not reach statistical significance when assessed with the Spearman correlation test. Moreover, no significant correlations with age, BMI, waist circumference, or blood pressure were noted (Table 4).

**Figure 1:**
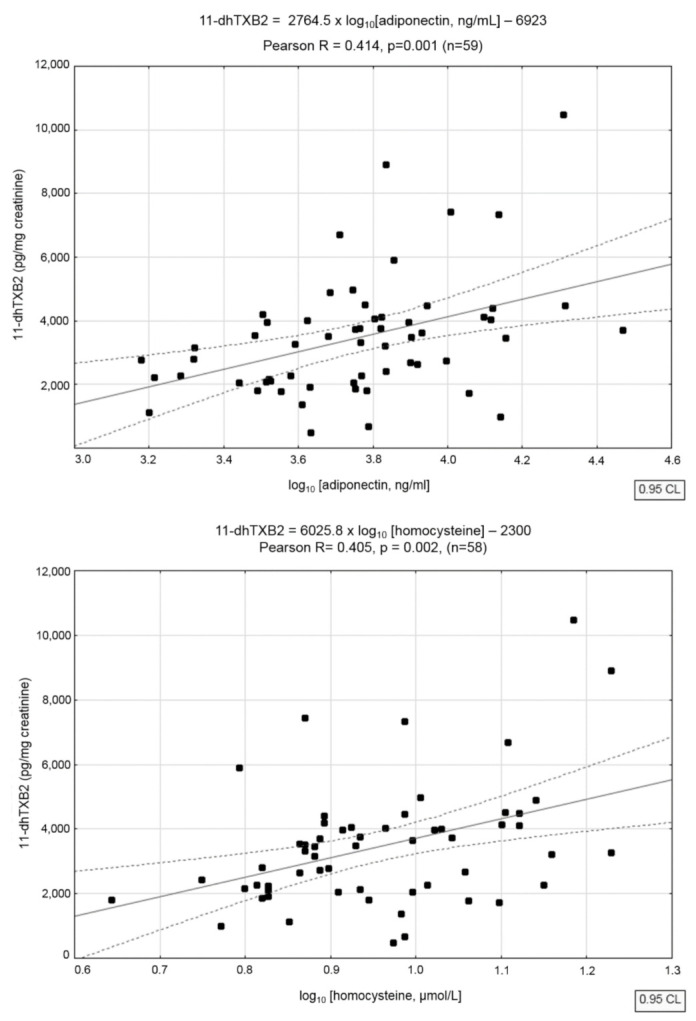

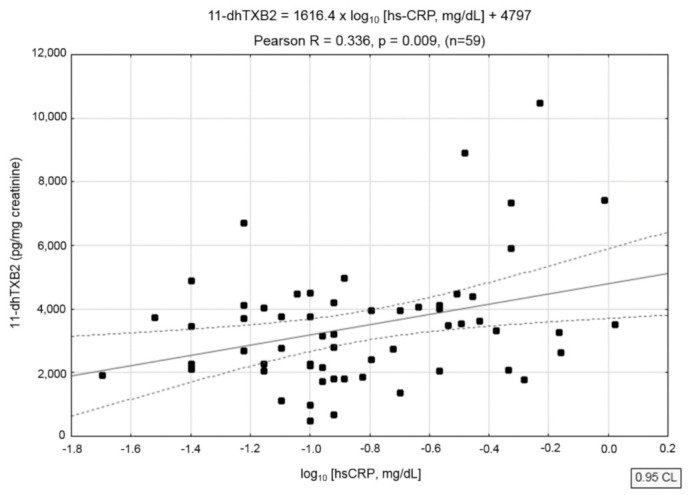
Correlation of urine 11-dehydro-TXB2 levels in aspirin- and statin-naive males with serum adiponectin (**upper chart**), homocysteine (**middle chart**), and hs-CRP (**lower chart**).

Correlations of 11-dehydro-TXB2 levels with lipids, hs-CRP, homocysteine, and adiponectin were assessed for the subgroup of patients not treated with statins, as statins might have affected these analytes. For the analytes with strongly skewed concentration frequency distributions, logarithmic transformation was used, and Pearson correlation coefficients were calculated. 11-dehydro-TXB2 levels were distinctly, positively, and significantly correlated with concentrations of serum adiponectin, homocysteine, and hs-CRP (Pearson R, after logarithmic transformation) (Figure 1).

Among the lipids, only LDL-C levels were significantly correlated with 11-dehydro-TXB2. Total cholesterol, non-HDL-cholesterol, and Apo B were also positively correlated with 11-dehydro-TXB2 (*p* ≈ 0.08), but the strength of these correlations did not attain statistical significance. No significant correlations with platelet count, insulin levels, HOMA-IR, glucose, or HbA1c were noted (Table 4).

The association between 11-dehydro-TXB2 and well-known laboratory markers of cardiovascular risk was also confirmed in additional analyses. 11-dehydro-TXB2 levels were higher in subjects with elevated homocysteine (>12 µmol/L; *p* = 0.009) and hs-CRP (>2 mg/L; *p* = 0.009) (Supplementary Table S2 and Figure S1).

### 3.4. Multiple Linear Regression Models

Parameters and analytes that correlated with 11-dehydro-TXB2 at the significance level *p* < 0.10 were included in multiple linear regression analyses. Concentrations of variables with log-normal distribution (i.e., hs-CRP, homocysteine, and adiponectin) were log-transformed before the calculations. The final results of the statistical analysis are shown in Table 5—homocysteine, adiponectin, and hs-CRP were found to be statistically significant predictors of 11-dehydro-TXB2 levels, with the explanatory power of the model given by Multiple R = 0.60 and R^2^ = 0.36. All tested models are presented in Supplementary Table S1.

## 4. Discussion

In the presented analysis, we revealed that increased levels of urine 11-dehydro-TXB2, an indirect measure of platelet activity, affect a large proportion of aspirin-naive men with MS. This biochemical marker was positively associated with two well-known cardiovascular risk factors, hs-CRP and homocysteine, as well as adiponectin. Our results substantiate the discussion regarding indications for aspirin use in some patients with MS, specifically when increased cardiovascular risk, estimated based on standard scores, coexists with unfavourable metabolic and prothrombotic profiles.

Urine 11-dehydro-TXB2 levels were elevated above the upper limit of the normal range for healthy people [21] in more than half of our patients with MS. This proportion increased to two-thirds when the data were interpreted using the algorithm proposed by Lopez et al., in which an 11-dehydro-TXB2 level of 2500 pg/mg creatinine was established as an upper normal limit for aspirin-naive healthy subjects [2]. The same level was adopted by Neath et al. to evaluate atherothrombotic risk [22].

Elevated urine 11-dehydro-TXB2 levels, a measure of native platelet activity, were previously reported in a large group of apparently healthy persons with MS and a family history of coronary artery disease and compared with those without MS, but the difference in the medians of those two subgroups was small (approximately 13%) [10]. More comprehensive studies on 11-dehydro-TXB2 as a marker of platelet activity, response to aspirin treatment, and prognostication were carried out in patients with diabetes and other cardiovascular diseases [2,23]. Lingling et al. reported a mean urine 11-dehydro-TXB2 level of 3436 pg/mg creatinine in a group of cerebral infarction patients (52 males and 65 females), which was practically the same as the levels reported in our patients [24]. They used the ELISA test to determine 11-dehydro-TXB2 and also adopted a cut-off of ≥2500 pg/mg creatinine for increased platelet activity. Increased platelet reactivity in MS has also been demonstrated by platelet function tests based on aggregometry (with a variety of agonists) [9].

The tests to quantify urine 11-dehydro-TXB2 were primarily developed to assess the adequacy of aspirin treatment, with the most frequent cut-off of 1500 pg/mg creatinine for the ELISA method [21]. Ten of our patients had urine 11-dehydro-TXB2 ≤ 1500 pg/mg creatinine as if they had already been on aspirin treatment. Therefore, they were not candidates for aspirin prevention. The same assessment was applied to all the patients with 11-dehydro-TXB2 levels < 2500 pg/mg creatinine. To make this algorithm [2] more precise, we assessed the ASCVD risk using the SCORE system to ensure the objectivity of evaluation in all our patients [14]. Eleven of our patients were high risk (SCORE ≥ 5%) and also had elevated 11-dehydro-TXB2 levels (among which three had a SCORE > 10%). In our opinion, such patients may benefit from low-dose aspirin for ASCVD prevention. Selak et al. conducted a study of 245,028 persons aged 30–79 years without established cardiovascular diseases and demonstrated a net benefit of aspirin treatment in 12.1% of men without known cardiovascular disease [25]. The benefit was more likely in subjects with higher levels of established cardiovascular disease risk factors. A recent meta-analysis of clinical outcomes of polypill use in primary prevention has shown a greater reduction in the primary endpoint (cardiovascular death, myocardial infarction, stroke, or arterial revascularisation) when the polypill contained aspirin in addition to antihypertensive medicines and statins [26].

In our subgroup of patients not treated with statins, multiple regression analysis revealed that the concentrations of homocysteine, adiponectin, and hs-CRP were predictors of 11-dehydro-TXB2 levels. Homocysteine is thought to influence platelet activity by increasing oxidative stress and causing endothelium dysfunction, which results in reduced nitric oxide (NO) synthesis and bioavailability [27]. The importance of oxidative stress for platelet activity has been indirectly shown, as its final product, 4-hydroxynonenal (4-HNE), is a predictor of aspirin resistance in patients with acute cerebral infarction [28]. Homocysteine is also a predictor of 11-dehydro-TXB2 levels in patients with arterial hypertension [29], which had a high prevalence in our group (93%). Increased platelet activity may be one of the indirect atherothrombotic effects of homocysteine [30]. Homocysteine also inhibits NO production in platelets [31]. Despite some doubts regarding the causative role of homocysteine in ASCVD and whether it is a risk factor, a risk marker, or just an epiphenomenon, Piazzolla et al. recently described epidemiological and clinical arguments that support homocysteine as an independent risk factor for atherosclerosis in patients with MS [32].

The positive association between 11-dehydro-TXB2 levels and adiponectin may plausibly be explained by its compensatory, alleviatory action in reducing oxidative stress, which enhances platelet activity. Adiponectin levels in almost all (90%) of our patients were within the typical range for healthy blood donors, except for eight patients with somewhat elevated levels. A counteracting effect of adiponectin on oxidative stress and inflammation may also explain the so-called adiponectin paradox (i.e., elevated levels of adiponectin in a variety of cardiovascular diseases and its association with mortality risk) [33,34]. Woodward et al. described a mechanism of increased adiponectin synthesis as a result of elevated oxidation products in the vascular wall, which diffuse to the perivascular adipose tissue, in turn increasing *ADIPOQ* gene expression and adiponectin release [35]. They described the mechanism as crosstalk between adipose tissue and vascular walls. Adiponectin is also increased in inflammatory and immune-mediated diseases [36]. The preincubation of platelets from MS patients with added adiponectin reduces their aggregatory response to epinephrine and adenosine diphosphate (ADP) [37]. Poor platelet responsiveness to aspirin is also significantly associated with increased levels of adiponectin in vitro [38].

Serum hs-CRP log-transformed concentrations were also correlated with 11-dehydro-TXB2 levels and were an independent predictor of these levels in our statin-naïve patients. Wang et al. reported that urinary 11-dehydro-TXB2 levels are associated with vascular inflammation (weakly with CRP and more strongly with circulating P-selectin and E-selectin) in patients with atherosclerotic cardiovascular disease [23]. The association of CRP levels with 11-dehyhdro-TXB2 has been previously studied, mainly in obese women. Urinary 11-dehydro-TXB2 levels were increased in obese females compared with those of normal weight [8]. 11-dehydro-TXB2 levels significantly increased in consecutive CRP quartiles. Weight loss resulted in a reduction in CRP and 11-dehydro-TXB2 levels [39]. CRP was also an independent predictor of platelet aggregation and aspirin non-responsiveness in patients with acute coronary syndrome complicated by pneumonia [40].

Among the assessed anthropometric measures, only WHR was a predictor of 11-dehydro-TXB2 in our male patients. The only similar association we found in the literature was that in obese women, WHR was a predictor of the 11-dehydro-TXB2 excretion rate, independent of adiponectin, CRP, CD40-L, and lipid patterns [41]. This may mean that the distribution of accumulated fat is more important for platelet activity than the total mass of adipose tissue.

Among the lipids, only the correlation of LDL cholesterol with 11-dehydro-TXB2 reached statistical significance (Table 4). Total cholesterol was a weak predictor of 11-dehydro-TXB2 levels, but reached statistical significance in the patients not treated with statins in one of the models of multiple regression analysis (Table S1). A statistically significant correlation between 11-dehydro-TXB2 excretion and total plasma cholesterol was found in patients with type IIa hypercholesterolemia [42]. LDL oxidation plays an important role in the complicated interaction between platelets and lipids in which oxidised LDLs (oxLDLs) activate platelets, and in turn, augment LDL oxidation [43]. In cases of hypercholesterolemia, increased LDL levels provide abundant lipid substrates for oxidation in the milieu of increased oxidative stress in MS.

We did not find an association between urine 11-dehydro-TXB2 levels and insulin or estimated insulin resistance (HOMA-IR). In normal subjects, insulin exerts an antiaggregatory impact on platelets [44]. However, in obese, insulin-resistant subjects, this effect was greatly reduced [45]. In our MS patients, the effect of somewhat increased insulin levels on platelet activity as assessed by 11-dehydro-TXB2 was probably offset by insulin resistance with no net result.

## 5. Clinical Implications

Despite the large proportion of men with MS and elevated 11-dehydro-TXB2, only some are at high risk of ASCVDs and may potentially benefit from personalised aspirin prevention, provided that they are not at increased risk of major bleeding. The accumulation of multiple ASCVD risk factors in some MS patients seems to justify this approach, despite the recent cautious guidance on the use of aspirin in primary prevention, which is generally not recommended. However, the authors of the 2021 ESC Guidelines on cardiovascular disease prevention in clinical practice “cannot exclude that in some patients at high or very high CVD risk, the benefits outweigh the risks” [7]. Measurements of 11-dehydro-TXB2 may be suggested, particularly in high-risk MS patients with elevated common routine parameters: hs-CRP, total, and LDL cholesterol, WHR, and even more so with increased homocysteine. Testing for 11-dehydro-TXB2, a marker of platelet activity, is non-invasive and inexpensive.

## 6. Limitations of the Study

The small sample size was the main limitation of our study. It was also a single-centre study so the results might not necessarily be generalisable to patient populations from other countries. Furthermore, our observations are restricted to men and cannot be extrapolated to women. The study group did not include very old subjects. It should also be noted that there are non-platelet sources of 11-dehydro-TXB2, which may contribute to its urine levels. Approximately 30% of systemic TXA2 production derives from, i.e., monocytes/macrophages and vascular endothelial cells. Non-platelet TXA2 biosynthesis increases in inflammatory conditions, also related to CVD [2,23,46]. Inflammatory cells produce TXA2 via the cyclooxygenase (COX) isoform COX-2, which is minimally affected by the low doses of aspirin used in clinical settings. Therefore, measurement of urinary levels of 11-dehydro-TXB2 has limited specificity for monitoring the effects of aspirin on platelets [47].

## 7. Conclusions

1. Increased levels of urine 11-dehydro-thromboxane B2 are frequent in aspirin-naive men with metabolic syndrome.
2. Urine 11-dehydro-TXB2 is correlated with well-known cardiovascular risk factors, specifically homocysteine, h-CRP, LDL cholesterol, and adiponectin.
3. Among males with MS, some with elevated urine 11-dehydro-TXB2 and a high cardiovascular risk may potentially benefit from aspirin primary prevention. Testing for 11-dehydro-TXB2 may help select patients for such prevention. Further studies are needed to prove this hypothesis.

## Figures and Tables

**Table 1:** Basic characteristics of the study group.

Variables	Mean ± SD	Median, Range
Age (years)	43.3 ± 9.7	44 (21.0–65.0)
WC (cm)	111.6 ± 8.9	113.0 (93.0–135.0)
BMI (kg/m^2^)	32.5 ± 3.7	31.2 (23.5–42.4)
WHR (-)	1.09 ± 0.05	1.09 (0.95–1.21)
SBP (mmHg)	143 ± 16.5	149 (110–180)
DBP (mmHg)	91 ± 10.3	90.0 (70–110)
PLT (10^3^/mm^3^)	216.4 ± 52.2	212.0 (134.0–366.0)
TG (mg/dL)	243.2 ± 124.7	210.0 (61.0–653.0)
HDL-C (mg/dL)	40.8 ± 8.6	40.5 (25.0–66.0)
TC (mg/dL)	220.8 ± 49.3	228.0 (107.0–342.0)
LDL-C (mg/dL)	131.4 ± 40.0	134.5 (28.0–234.0)
Non-HDL-C (mg/dL)	261.6 ± 52.9	269.0 (134.0–389.0)
ApoB (mg/dL)	109.0 ± 26.3	111.0 (53.0–174.0)
FG (mg/dL)	99.7 ± 9.5	99.0 (82.0–125.0)
HbA1c (%)	5.81 ± 0.37	5.75 (5.10–6.80)
Creatinine (mg/dL)	0.89 ± 0.13	0.90 (0.60–1.30)
eGFR (mL/min/1.73 m^2^)	102.4 ± 17.5	105.5 (65.0–157.0)
Adiponectin (ng/mL)	7341 ± 5049	6014 (1507–29456)
Insulin (µU/mL)	16.20 ± 9.53	13.6 (3.71–55.13)
HOMA-IR	4.05 ± 2.47	3.26 (0.89–13.07)
hs-CRP (mg/L)	2.33 ± 2.36	1.30 (0.10–10.5)
Homocysteine (µmol/L)	9.72 ± 2.94	9.30 (4.40–20.3)

Apo—apolipoprotein B, BMI—body mass index, DBP—diastolic blood pressure, eGFR—estimated glomerular filtration rate, FG—fasting glucose, HbA1C—glycated haemoglobin, HDL-C—high-density lipoprotein cholesterol, hs-CRP—high-sensitivity C-reactive protein, HOMA-IR—homeostatic model assessment (estimated insulin resistance), LDL-C—low-density lipoprotein cholesterol, Non-HDL-C—non-HDL cholesterol, PLT—platelet count, SBP—systolic blood pressure, TC—total cholesterol, TG—triglycerides, WC—waist circumference, WHR—waist-to-hip ratio.

**Table 2:** Distribution of patients’ cardiovascular risk (SCORE) [14].

ESC SCORE	No of the Patients	Proportion (%)
<1%	10	12.2
≥1% and <5%	56	68.3
≥5% and <10%	12	14.6
≥10%	4	4.9

**Table 3:** Urinary 11-dehydro-thromboxane B2 (11-dehydro-TBX2) levels in the study group with stratification according to the algorithm by Lopez et al., 2014 [2].

11-Dehydro-TBX2 pg/mg Creatinine	No. of Patients	Mean ± SD pg/mg Creatinine	Range	ESC SCORE ≥ 5% No. of Patients
11-dhTXB2 (all)	82 (100%)	3453 ± 2010	494–12,224	16
11-dhTXB2 < 2500 *	27 (32.9%)	1668 ± 606	494–2433	5
2500 ≤ 11-dhTXB2 ≤ 10,000	53 (64.6%)	4064 ± 1283	2583–8927	10
TBX > 10,000	2 (2.4%)	11,353 ± 1231	10,483–12,224	1

* including 10 patients with 11-dhTBX2 ≤ 1500 ng/mg creatinine. Mean 11 dehydro-TxB2 in healthy individuals: 1119 pg/mg creatinine, range: 62–3121 [21].

**Table 4:** Correlation (Spearman R) of 11-dehydro-TXB2 with anthropometric, blood pressure, and laboratory measures.

Variables	*N*	Spearman R	*p*
Age (years)	82	0.080	0.479
WC (cm)	82	0.080	0.479
BMI (kg/m^2^)	82	0.080	0.478
WHR (-)	82	0.199	0.074
SBP (mmHg)	82	−0.039	0.726
DBP (mmHg)	82	0.062	0.577
PLT (10^3^/mm^3^)	81	0.049	0.665
TG (mg/dL)	59	−0.025	0.848
HDL-C (mg/dL)	59	0.010	0.938
TC (mg/dL)	59	0.229	0.081
LDL-C (mg/dL)	59	0.262	0.044
Non-HDL-C (mg/dL)	59	0.227	0.083
ApoB (mg/dL)	59	0.227	0.084
FG (mg/dL)	82	0.014	0.902
HbA1c (%)	82	−0.042	0.709
Insulin (µU/mL)	78	0.035	0.756
HOMA-IR	78	0.032	0.783

ApoB—apolipoprotein B, BMI—body mass index, DBP—diastolic blood pressure, FG—fasting glucose, HbA1c—glycated haemoglobin, HDL-C—high-density lipoprotein cholesterol, HOMA-IR—homeostatic model assessment (estimated insulin resistance), LDL-C—low-density lipoprotein cholesterol, Non-HDL-C—non-HDL cholesterol, PLT—platelet count, SBP—systolic blood pressure, TC—total cholesterol, TG—triglycerides, WC—waist circumference, WHR—waist-to-hip ratio.

**Table 5:** Multiple linear regression analysis for correlates of 11-dehydro-thromboxane B2.

Explanatory Variables	β ± SE	*p*
Log_10_ (Homocysteine)	0.3353 ± 0.1111	0.0039
Log_10_ (Adiponectin)	0.3081 ± 0.1119	0.0080
Log_10_ [hs-CRP)	0.2924 ± 0.1096	0.0100
Model 3 summary: Multiple R = 0.6017, R^2^ = 0.3621, F (3.54) = 10.271, *p* < 0.00002		

F—F-statistic, hs-CRP—high-sensitivity C-reactive protein, *p*—significance level, Multiple R—multiple correlation coefficient, R^2^—determination coefficient, SE—standard error, β—standardised regression coefficient.

## Data Availability

The data presented in this study are available on request from the corresponding author. The data are not publicly available due to internal institutional rules.

## Supplementary Materials

The following supporting information can be downloaded at: https://www.mdpi.com/article/10.3390/jcm11123471/s1, Table S1. Multiple linear regression analysis for correlates of 11-dehydro-TxB2-3 tested models. When hs-CRP, WHR, non-HDL-C, LDL-C, apoB, and TC were included in the model, only hs-CRP, WHR, and TC were independent, statistically significant predictors of 11-dehydro-TXB2 levels (Model 1). When additionally homocysteine was included in the analysis, only homocysteine and hs-CRP retained statistical significance, homocysteine being the stronger predictor (Model 2). When adiponectin was included in multiple regression calculations, three analytes—homocysteine, adiponectin, and hs-CRP—remained statistically significant predictors (Model 3). Table S2. 11-dehydro-TXB2 levels below and above the chosen cut-offs (the upper limit of the reference range (for homocysteine 12 µmol/L), the limit for the increased risk of CVD (hs-CRP (2 mg/L), the median value of WHR (1.08). Figure S1 Urine 11-dehydro-TXB2 levels in aspirin and statin naïve males with metabolic syndrome stratified into different levels of predictors (normal and elevated): homocysteine (a), hs-CRP (b), and waist-to-hip ratio (below and above the median) (c).
