# Supplementary material for: Urine 11-Dehydro-Thromboxane B2 in Aspirin-Naive Males with Metabolic Syndrome
Source: J Clin Med. 2022 Jun 16;11(12):3471. doi: 10.3390/jcm11123471 (PMC9224828; doi:10.3390/jcm11123471)
Supplement: Supplementary file 1 [file jcm-11-03471-s001.zip › jcm-1758369-supplementary.pdf]

Supplementary Table S1. Multiple linear regression analysis for correlates of 11-dehydro-TxB2 - 3 tested models. When hs-CRP, WHR, non-HDL-C, LDL-C, apoB, and TC were included in the model only hs-CRP, WHR, and TC were independent, statistically significant predictors of 11-dehydro-TXB2 levels (Model 1). When additionally homocysteine was included in the analysis, only homocysteine and hs-CRP retained statistical significance, homocysteine being the stronger predictor (Model 2). When adiponectin was included in multiple regression calculations three analytes: homocysteine, adiponectin, and hs-CRP remained statistically significant predictors (Model 3).

Explanatory variables	β ± SE	p
Model 1		
Log_{10} [hs-CRP]	0.3715 ± 0.1181	0.0027
WHR	0.2999 ± 0.1182	0.0141
TC	0.2555 ± 0.1181	0.0350
Model 1 summary: Multiple R = 0.5038, R^2 = 0.25380, F (3.54) = 6.1238, p < 0.0012		
Model 2		
Log_{10} [Homocysteine]	0.3982 ± 0.1150	0.0010
Log_{10} [hs-CRP]	0.3301 ± 0.1150	0.0058
Model 2 summary: Multiple R = 0.5221, R^2 = 0.2726, F (2.55) = 10.3062, p < 0.0002		
Model 3		
Log_{10} [Homocysteine]	0.3353 ± 0.1111	0.0039
Log_{10}[Adiponectin]	0.3081 ± 0.1119	0.0080
Log_{10} [hs-CRP]	0.2924 ± 0.1096	0.0100
Model 3 summary: Multiple R = 0.6017, R^2 = 0.3621, F (3.54) = 10.271, p < 0.00002		
F – F-statistic, hs-CRP - high-sensitivity C-reactive protein, p – significance level, R - multiple correlation coefficient, R^2 – determination coefficient, SE – standard error, WHR – waist to hip ratio, β – standardized regression coefficient.		

Supplementary Table S2. 11-dehydro-TXB2 levels below and above the chosen cut-offs (upper limit of the reference range (for homocysteine 12 µmol/L), the limit for the increased risk of CVD (hs-CRP (2 mg/L), the median value of WHR (1.08)

	HCY<12 µmol/L N=46 Mean±SD	HCY>12 µmol/L N=12 Mean±SD	P*	hs-CRP≤2 mg/L N= 39 Mean±SD	hs-CRP>2 mg/L N= 20 Mean±SD	P*	WHR<1.08# N=24 Mean±SD	WHR≥1.08 N=34 Mean±SD	P*
11-dhTXB2 pg/mg creatinine	3080 ± 1488	4900 ± 2603	0.009	2912 ± 1332	4533 ± 2336	0.009	2779 ± 1118	3994 ± 2155	0.024

HCY – homocysteine, hs-CRP - high-sensitivity C-reactive protein, WHR – waist to hip ratio,

* Mann-Whitney test, #Median for 58 patients

Figure S1. Urine 11-dehydro-TXB2 levels in aspirin and statin naïve males with metabolic syndrome stratified into different levels of the predictors (normal and elevated, cut-off – median value): homocysteine (upper chart), hs-CRP (middle chart), and waist to hip ratio (lower chart).

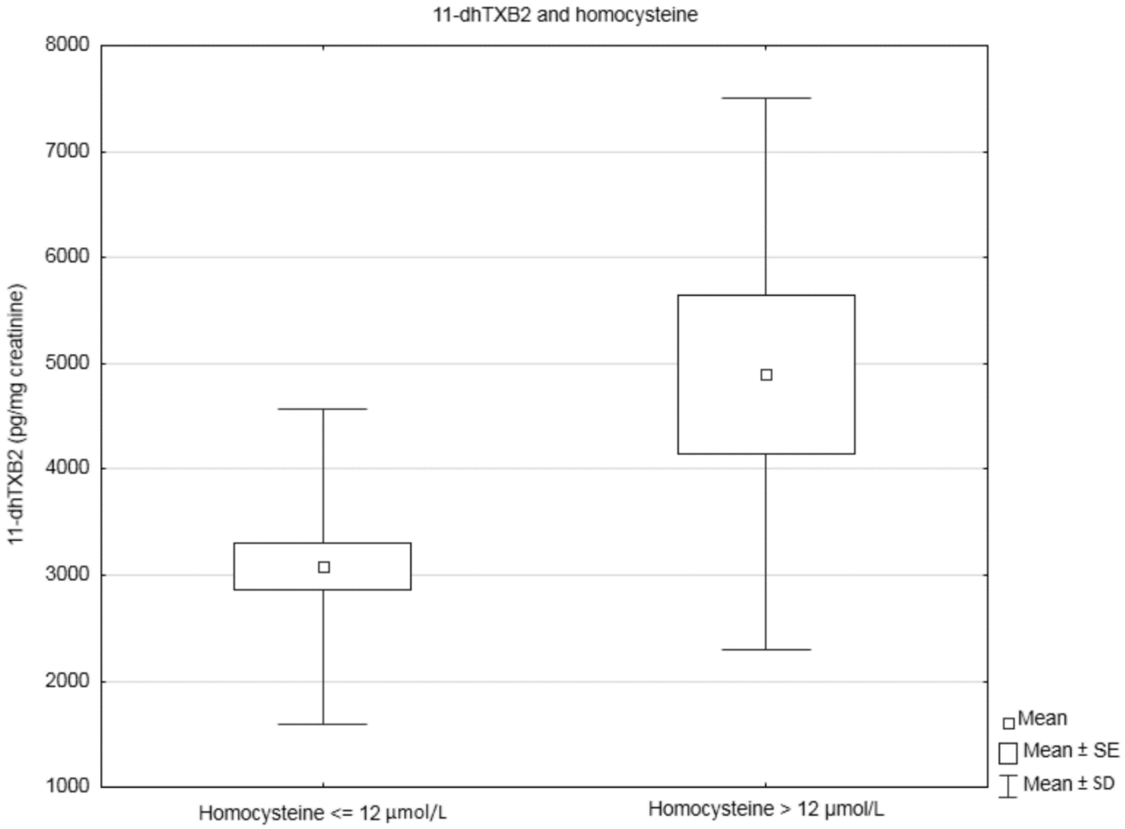

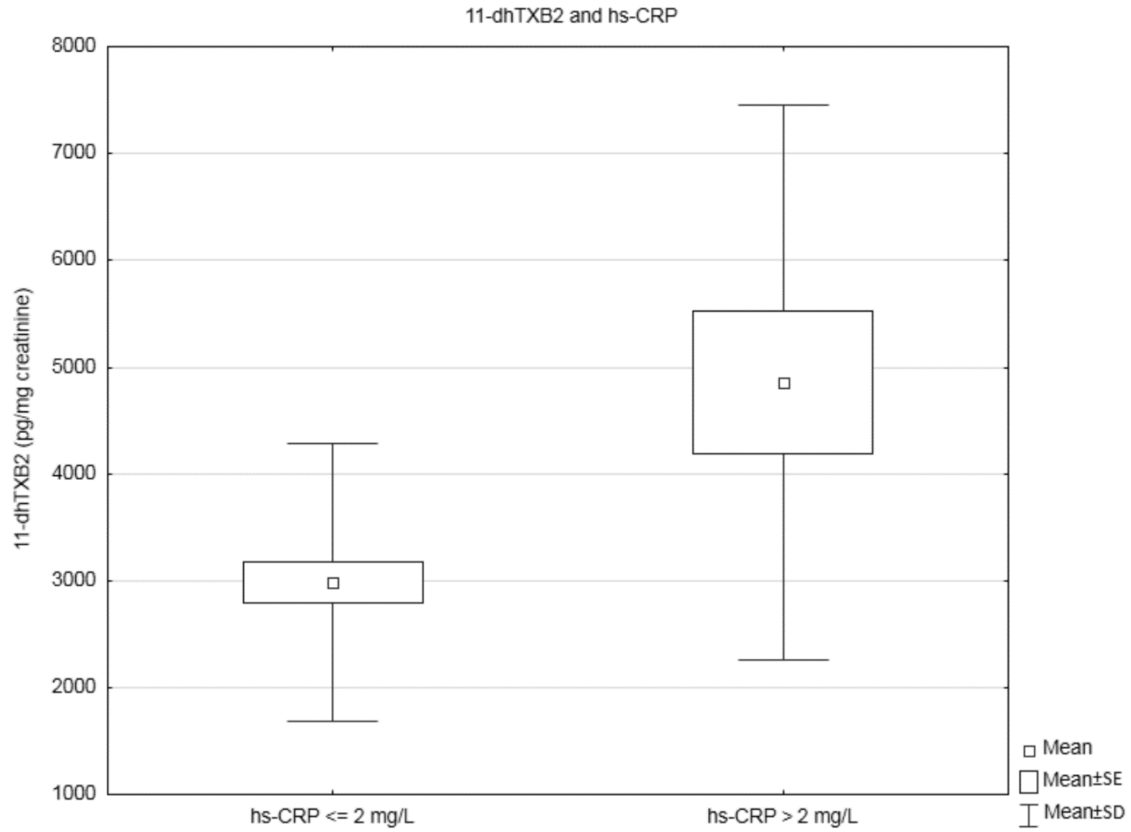

11-dhTXB2 and hs-CRP
11-dhTXB2 (pg/mg creatinine)
8000
7000
6000
5000
4000
3000
2000
1000
hs-CRP <= 2 mg/L
hs-CRP > 2 mg/L
Mean
Mean±SE
Mean±SD

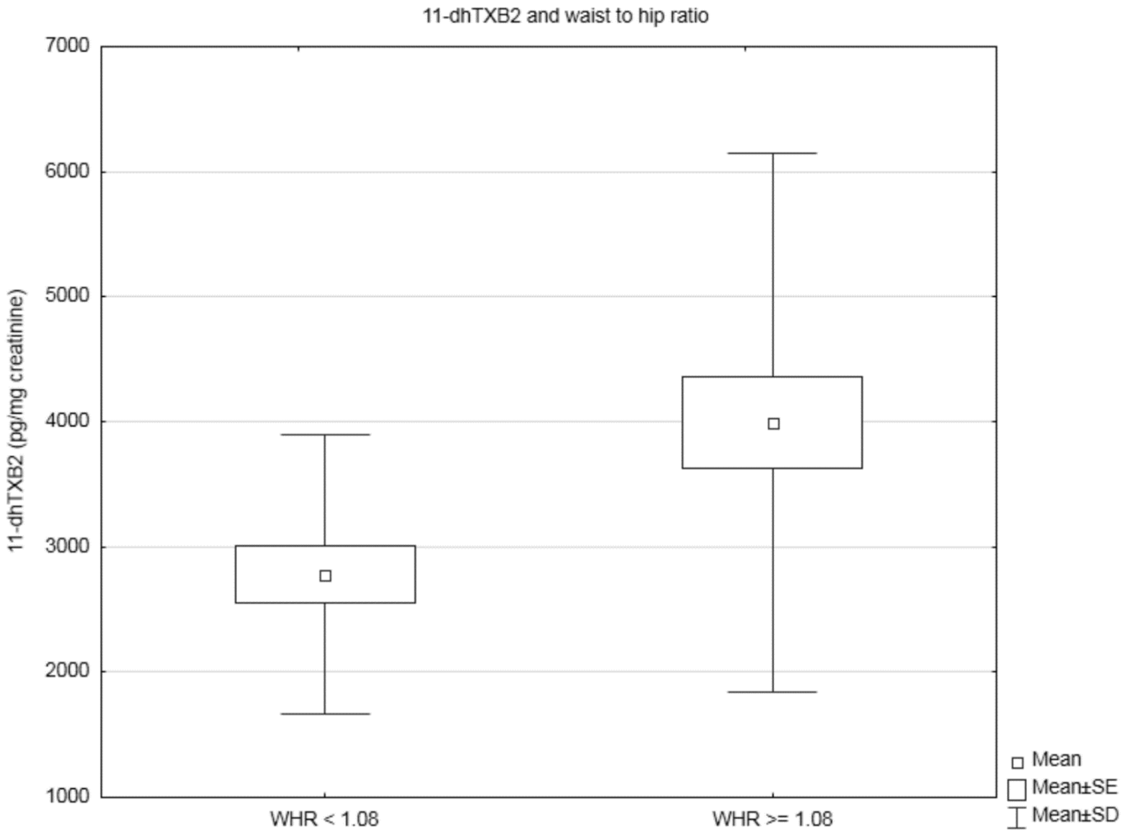
11-dhTXB2 and waist to hip ratio
11-dhTXB2 (pg/mg creatinine)
7000
6000
5000
4000
3000
2000
1000
WHR < 1.08
WHR >= 1.08
Mean
Mean±SE
Mean±SD
